# Monitoring European data with prospective space–time scan statistics: predicting and evaluating emerging clusters of COVID-19 in European countries

**DOI:** 10.1186/s12889-022-14298-z

**Published:** 2022-11-25

**Authors:** Mingjin Xue, Zhaowei Huang, Yudi Hu, Jinlin Du, Miao Gao, Ronglin Pan, Yuqian Mo, Jinlin Zhong, Zhigang Huang

**Affiliations:** 1 Guangdong Medical University, Zhanjiang, Guangdong Province China; 2 Pension Industry Research Institute, Guangdong Medical University, Guangdong Province Zhanjiang, China

**Keywords:** COVID-19, Space–time clusters, Predict, STSS, Monitor

## Abstract

**Background:**

Coronavirus disease 2019 (COVID-19) has become a pandemic infectious disease and become a serious public health crisis. As the COVID-19 pandemic continues to spread, it is of vital importance to detect COVID-19 clusters to better distribute resources and optimizing measures. This study helps the surveillance of the COVID-19 pandemic and discovers major space–time clusters of reported cases in European countries. Prospective space–time scan statistics are particularly valuable because it has detected active and emerging COVID-19 clusters. It can prompt public health decision makers when and where to improve targeted interventions, testing locations, and necessary isolation measures, and the allocation of medical resources to reduce further spread.

**Methods:**

Using the daily case data of various countries provided by the European Centers for Disease Control and Prevention, we used SaTScan™ 9.6 to conduct a prospective space–time scan statistics analysis. We detected statistically significant space–time clusters of COVID-19 at the European country level between March 1st to October 2nd, 2020 and March 1st to October 2nd, 2021. Using ArcGIS to draw the spatial distribution map of COVID-19 in Europe, showing the emerging clusters that appeared at the end of our study period detected by Poisson prospective space–time scan statistics.

**Results:**

The results show that among the 49 countries studied, the regions with the largest number of reported cases of COVID-19 are Western Europe, Central Europe, and Eastern Europe. Among the 49 countries studied, the country with the largest cumulative number of reported cases is the United Kingdom, followed by Russia, Turkey, France, and Spain. The country (or region) with the lowest cumulative number of reported cases is the Faroe Islands. We discovered 9 emerging clusters, including 21 risky countries.

**Conclusion:**

This result can provide timely information to national public health decision makers. For example, a country needs to improve the allocation of medical resources and epidemic detection points, or a country needs to strengthen entry and exit testing, or a country needs to strengthen the implementation of protective isolation measures. As the data is updated daily, new data can be re-analyzed to achieve real-time monitoring of COVID-19 in Europe. This study uses Poisson prospective space–time scan statistics to monitor COVID-19 in Europe.

## Background

Coronavirus disease 2019 (COVID-19), which is caused by the highly pathogenic virus severe acute respiratory syndrome coronavirus 2 (SARS-CoV-2), was first detected in Wuhan, China, in December 2019 and has since become a pandemic infectious disease also become a serious public health crisis [1]. As recognized by the World Health Organization(WHO), the use of mathematical methods to establish a dynamic spread model of infectious diseases in the early stages of an infectious disease epidemic plays a key role in providing decision-makers based on data evidence. At present, the COVID-19 pandemic has promoted the unprecedented development of infectious disease transmission dynamics models and incorporated them into policy formulation and public health practices [2]. These infectious disease transmission dynamics model provides a scientific method to study the dynamics of disease transmission and to derive long-term and short-term predictions. These predictions clearly integrate assumptions about the epidemiological process affecting disease transmission and surveillance. During the outbreak of the COVID-19 pandemic, transmission dynamics models are very valuable. It can identify possible trends in the development of the disease, evaluate the effectiveness of the interventions, and predict the extent of spread of the disease [2].

Surveillance of space–time clusters of cases is one of the main ways to detects outbreaks of infectious diseases [3]. During the period of emerging infectious diseases such as COVID-19, the implementation of space–time monitoring is crucial, which can predict emerging clusters in advance, implement targeted intervention measures, early detection, and medical resource allocation. Space time scan statistics(STSS) is a method proposed by Kulldorff [4] to quickly monitor disease clusters based on scan statistics and find high-risk areas in advance. STSS are widely used in the monitoring of major infectious diseases. It can study the areas of high or low aggregation of diseases, and choose different data models to determine whether the space–time distribution of the observed diseases is accidental or random. To put it simply, it uses scan statistics to detect clusters of outliers (eg, outliers outside of a given baseline condition). This scan statistic uses a moving cylinder to scan the area, looking for potential space–time clusters of cases [4]. The bottom of the cylinder is the space scanning window, and the height reflects the time scanning window. The center of the cylinder is defined as the geographic coordinates of the center of each region. For example, if the number of cases in the space–time clusters scanned exceeds 50% of the population at risk, it indicates that the outside of the scanning cylinder is a low-risk area. In its scanning cylinder, the results will show the location, size and duration of statistically significant cluster disease cases.

In order to routinely monitor the epidemic, prospective space–time scan statistics [5] is a method of detecting "active" or emerging disease clusters, which can be used to monitor ongoing epidemics. Scan statistics will detect clusters that are "active" at the end of the study period. The main purpose of using prospective scan statistics instead of retrospective scan statistics is to only focus on the significant clusters that are "active" or that exist at the time of analysis. It ignores the clusters that may have existed before are no longer a threat to the public health neighborhood [5]. For instance, prospective space–time scan statistics have been used to detect Shigellosis [6], measles [7], syndrome surveillance [8], and recently COVID-19 [9–11]. The results indicate that prospective scanning is a tool that low-income and middle-income countries can use to detect emerging clusters and implement specific control policies and interventions to slow the spread of COVID-19 [12]. Since COVID-19 data is updated daily, prospective space–time scan statistics can help to monitor the pandemic in time, and the focus in this study is on Europe.

This study helps the surveillance of the COVID-19 pandemic and discovers major space–time clusters of reported cases in European countries. Prospective space–time scan statistics are particularly valuable because it has detected active and emerging COVID-19 clusters [13]. It can prompt public health decision makers when and where to improve targeted interventions, testing locations, and necessary isolation measures, and the allocation of medical resources to reduce further spread. In order to prove the effectiveness of using prospective space–time scan statistics, we report the results of two time periods: March 1, 2020 to October 2, 2020 and March 1, 2021 to October 2, 2021. Compare the statistical results of prospective scans in Europe in 2020 with the results of actual risk areas in Europe in 2021, evaluate the effect of prospective space–time scan statistics, and propose clusters of emerging clusters that we have discovered. Since COVID-19 is a highly infectious disease that all people are susceptible to, we decided not to adjust for age. However, infants, young children, the elderly, and people with a previous medical history accounted for the vast majority of deaths from COVID-19, which can be corrected using the age-adjusted Bernoulli model, but this is not within the scope of this study.

## Methods

### Data sources

We collected COVID-19 case and population data from the European Center for Disease Control and Prevention. These data can be obtained for free on the page (https://www.ecdc.europa.eu/en/cases-2019-ncov-eueea). For the time being, these data are currently updated daily, and we are using the data available between March 1, 2020 to October 2, 2020 and March 1, 2021 to October 2, 2021. From a spatial perspective, if COVID-19 is clustered at the national level, the number of confirmed cases per day will be used for scanning statistics.

**Fig. 1:**
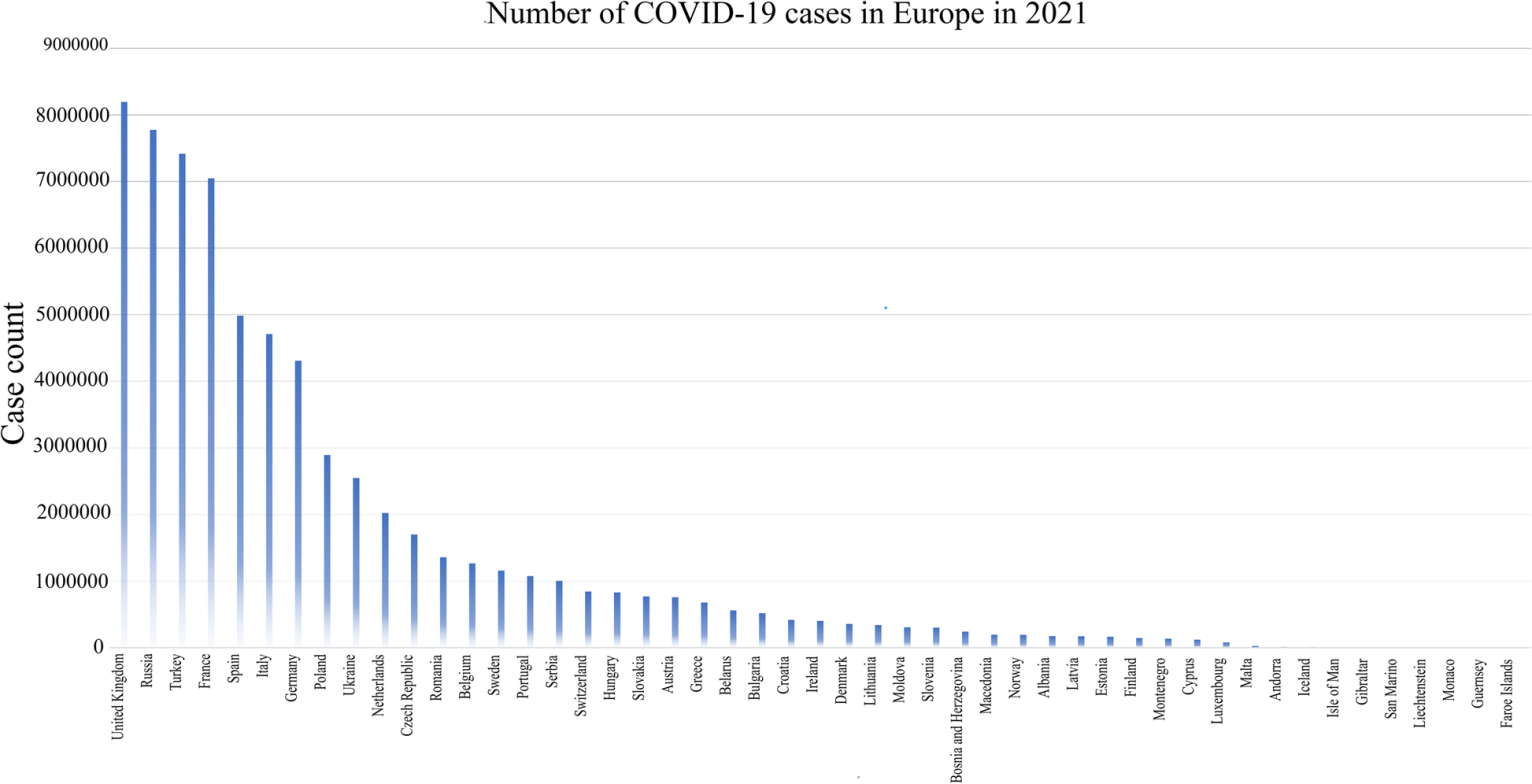
Cumulative number of COVID-19 cases in European countries between March 1st and October 2nd, 2021 (used for the statistical analysis)

Using the spatial location information in the COVID-19 dataset and the geographic information we obtained on Google Maps, we matched the geographic location information of the corresponding country to the case dataset. Our analysis focuses on 30 countries in the European Union and 19 countries outside the European Union, excluding some cases in European island countries and very small populations (without information). The COVID-19 data set of 49 countries(For each country code, see Table 1) reported the number of daily cases, so we can directly use the case data of each day (with missing values, you can query the daily cumulative number of cases announced by the WHO. The number of cases in the previous day (N_k_ -1) subtract from the number of cases (N_k_) on the day, so that you can get the number of new cases). The COVID-19 data set reports the cumulative number of cases in each country from March 1st to October 2nd, 2021 (Fig. 1).

**Table 1:** Assignment table of each country code

ID	Name	ID	Name
1	Albania	26	Liechtenstein
2	Andorra	27	Lithuania
3	Austria	28	Luxembourg
4	Belarus	29	Malta
5	Belgium	30	Moldova
6	Bosnia and Herzegovina	31	Monaco
7	Bulgaria	32	Montenegro
8	Croatia	33	Netherlands
9	Cyprus	34	Macedonia
10	Czech Republic	35	Norway
11	Denmark	36	Poland
12	Estonia	37	Portugal
13	Faroe Islands	38	Romania
14	Finland	39	Russia
15	France	40	San Marino
16	Germany	41	Serbia
17	Gibraltar	42	Slovakia
18	Greece	43	Slovenia
19	Guernsey	44	Spain
20	Hungary	45	Sweden
21	Iceland	46	Switzerland
22	Ireland	47	Turkey
23	Isle of Man	48	Ukraine
24	Italy	49	United Kingdom
25	Latvia

### Statistical analysis

#### Poisson prospective space–time scan statistics

Space–time scan statistics is an extension of space scan statistics proposed by Professor Kulldorff of Harvard Medical School in 1997. It adds a time dimension to the original space scan statistics, so that the scan statistics can detect clusters in both time and space. In order to identify the space–time clusters that are still occurring or "active", we use a Poisson prospective space–time scan statistics [5, 14, 15], and in SaTScan™ 9.6 realization(The parameters are shown in Table 2). Compared with the circular window of space scan statistical data, the space–time scan window has also become a cylinder correspondingly. The size of the scanning window of the cylinder corresponds to the spatial range, and the height corresponds to the time. The size and position of the scanning window of the cylinder change all the time, so that the space–time scan statistics can be used to determine the time and place of the epidemic. In-depth analysis of the size and scale of the gathering point, so as to realize the early recognition of the outbreak.

**Table 2:** Parameters used for the Prospective STSS analysis

Years	2020	2021
Probability Model	Discrete Poisson	Discrete Poisson
Spatial window shape	Circular	Circular
Maximum Spatial window area	20% of the population at risk	10% of the population at risk
Minimum Temporal cluster duration	14 days	14 days
Maximum Temporal cluster duration	50% of the study period	50% of the study period
Maximum Monte Carlo permutations	999	999
*P*-value significance	*p*-value < 0.05	*p*-value < 0.05

The process of space–time scan statistics includes the following four aspects. First, set a coordinate point in the study area as the center of the scanning window on the bottom of the cylinder. Second, gradually increase the radius and height of the bottom surface of the cylindrical scanning window until the time and space constraints of the maximum scanning window are reached. Repeat the same scanning process for all positions of the cylinder scanning window in the study area. Third, the expected number of cases can be calculated based on the number of observed cases inside the scanning cylinder and outside the scanning cylinder, the expected incidence rate can be calculated based on the number of observed cases and the number of people, and the incidence period can be calculated according to the selection of scanning frequencies at different times. The log likelihood ratio(LLR) of the test statistics is composed of the actual incidence and the expected incidence; LLR is used to evaluate the degree of abnormality in the number of cases in the scan window. The larger the log-likelihood ratio, the degree of abnormal disease in the scan window Bigger. Finally, a standard Monte Carlo simulation method is used to evaluate the statistical significance of the scanned cylinder.

We assume that COVID-19 cases follow a Poisson distribution according to the population of the geographic area. Null hypothesis H_0_: The risk of COVID-19 within the scanning area is the same as that outside the scanning area, and the intensity μ is proportional to the population at risk. Alternative Hypothesis H_1_: The risk of COVID-19 in the scanning cylinder is higher. The expected number of COVID-19 cases (μ) under the null hypothesis H_0_ is shown in Eq. (1):

1 $$\mu =p*C/P$$

where p represents the population in the scanning cylinder, C represents the total number of cases, and P represents the total population. The log-likelihood ratio is used to identify the window of outliers (high risk) in COVID-19 scanning, and it is defined as Eq. (2):

2 $$LLR=\frac{{L}_{Z}}{{L}_{0}}=\frac{{\left(\frac{{N}_{Z}}{{\upmu }_{Z}}\right)}^{{N}_{Z}}{\left(\frac{{N}_{T}-{N}_{Z}}{{\mu }_{T}-{\mu }_{Z}}\right)}^{{N}_{T}-{N}_{Z}}}{{\left(\frac{{N}_{T}}{{\mu }_{T}}\right)}^{{N}_{T}}}$$

where L_Z_ is the likelihood function of the scanning cylinder Z, L_0_ is the likelihood function of the cylinder H_0_; μ_Z_ is the expected number of events in the scanning cylinder Z; μ_T_ is the total expected number of theoretical events in the entire research space–time range: ${\mu }_{T}=\sum {\mu }_{Z}$; N_T_ is the total number of COVID-19 cases observed in Europe during the study period. N_Z_ is the number of COVID-19 cases observed in scanning cylinder Z. When the likelihood ratio is greater than 1, the risk of scanning the cylinder increases, that is: $\frac{\mathrm{NZ}}{\mathrm{\mu Z}}>\frac{{\mathrm{N}}_{\mathrm{T}}-{\mathrm{N}}_{\mathrm{Z}}}{{\mathrm{N}}_{\mathrm{T}}-{\upmu }_{\mathrm{Z}}}$.

In order to avoid the assumption that the relative risk of COVID-19 is homogeneous in a significant space–time cluster, we also report and visualize the relative risk of each country belonging to the cluster. From Eq. (3), the relative risk (RR) of each position in the cluster can be obtained:

3 $$RR=\frac{{\mathrm{N}}_{\mathrm{Z}}/{\upmu }_{\mathrm{Z}}}{({\mathrm{N}}_{\mathrm{T}}-{\mathrm{N}}_{\mathrm{Z}})/({\upmu }_{\mathrm{T}}-{\upmu }_{\mathrm{Z}})}$$

where N_Z_ is the total number of COVID-19 cases in a country, μ_Z_ is the expected number of cases in a country, N_T_ is the total number of cases observed in Europe. RR is the estimated risk within a location divided by the risk outside the location (ie, other locations). For example, if a country’s RR is 3, then the population of that country will be three times more likely to be exposed to COVID-19. The reported clusters also have relative risks, which are derived in the same way as Eq. (3); but the RR of the cluster is the estimated risk (observed value/expected value) within the cluster divided by the risk outside the cluster.

We define the scanning time in days as the unit, and the scanning area in the country as the unit. In order to avoid very large clusters, we used 2020 data to try 5, 10, 15, 20, 25, and 50% of high-risk populations as spatial scanning windows. When 5%, 15%, 25%, and 50% of high-risk populations are used as the maximum scanning window [16], the number of cities covered by certain clusters exceeds 30% of the total number of geographic countries (> 14 countries), which is not suitable or not conducive to disease monitoring [17]. In other words, the total number of clusters calculated by scanning has covered 90% of geographic countries. Therefore, under comprehensively weighing the accuracy of clustering and the actual situation of disease monitoring, the maximum spatial scanning area analyzed in 2020 is set to 20% of the population at risk, and the maximum spatial scanning area analyzed in 2021 is set to 10% of the population at risk. The other settings are the same. The maximum temporal cluster duration is set to 50% of the total study duration, the minimum temporal cluster duration is set to two longest incubation periods (14 days), the minimum number of cases is set to 5 cases, and the number of Monte Carlo iterations is set to 999 times. The space–time scan analysis adopts the Poisson probability model. According to the Poisson distribution principle, the LLR of different windows is calculated, and the Monte Carlo method is used for testing to evaluate the statistical significance of the space–time clusters. When *P* < 0.05, it can be considered that the relative risk of cases inside the window and the relative risk of cases outside the window are statistically significant. The area with the largest LLR value is regarded as the main cluster, and the other areas with statistically significant LLR values ​​are regarded as the secondary clusters. Use ArcGIS™ 10.2 to visualize the results of space–time scanning.

#### Root mean square error

The expected value predicted by the model is compared with the actual value to judge the prediction effect. We use the root mean square error (RMSE) method to analyze. The RMSE represents the distance between the expected value and the true value. It is the square root of the deviation between the observed value and the true value and the square root of the ratio of the number of observations N. In actual measurement, the number of observations N is always limited, and the true value can only be Replace with the most reliable (best) value (Eq. 4). RMSE is very sensitive to very large or very small errors in a set of measurements, so RMSE is a good indicator of the precision of a measurement, which is why RMSE is widely used. Therefore, this method was adopted in our study.

4 $$RMSE=\sqrt{\frac{{\sum }_{i=1}^{N}{\left({x}_{i}-{\widehat{x}}_{i}\right)}^{2}}{N}}$$

where RMSE means root mean square error; *i* means variable i; *N* means number of non-missing data points; *x*_*i*_ means actual observations time series; ${\widehat{x}}_{i}$ means estimated time series; a range of RMSE values not exceeding 2 is reasonable.

## Results

### The spatial distribution of the European population

**Fig. 2:**
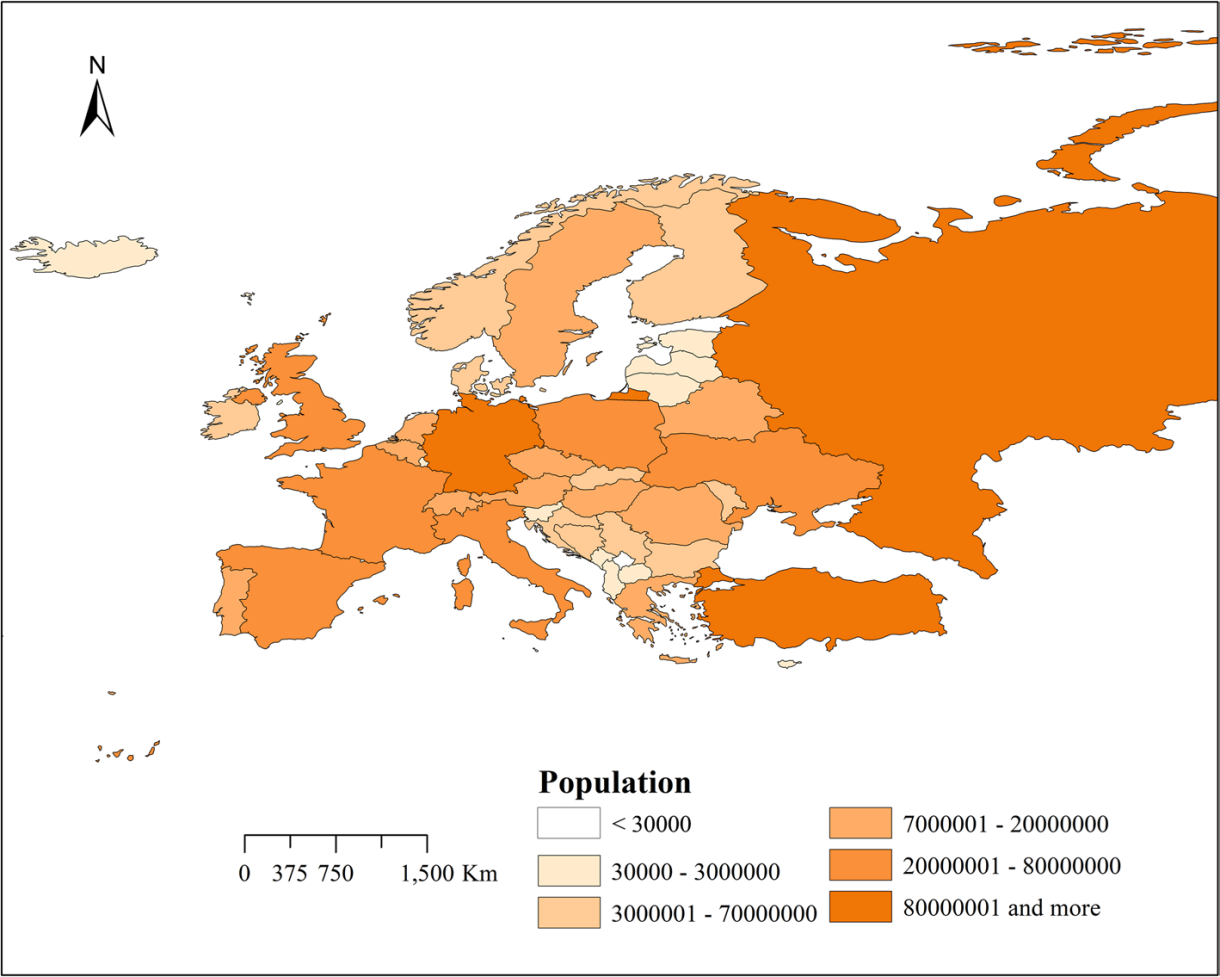
The spatial distribution map of the population of various countries in the European region between March 1st and October 2nd, 2021(data as of the end of 2019)

Figure 2 shows the spatial distribution of the population of 49 countries studied in Europe from March 1,2021 to October 2, 2021 (as of the end of 2019). There are 6 levels in total. The top 3 countries (or regions) in population are Russia, Germany, and Turkey, and the least populated region is Gibraltar. The population of Northern Europe is relatively small. Western Europe has the densest population distribution, followed by Central Europe. Because Eastern Europe is located at the junction of the Eurasian plates, the population is also densely distributed.

### The spatial distribution of the cumulative number of COVID-19 cases in Europe

**Fig. 3:**
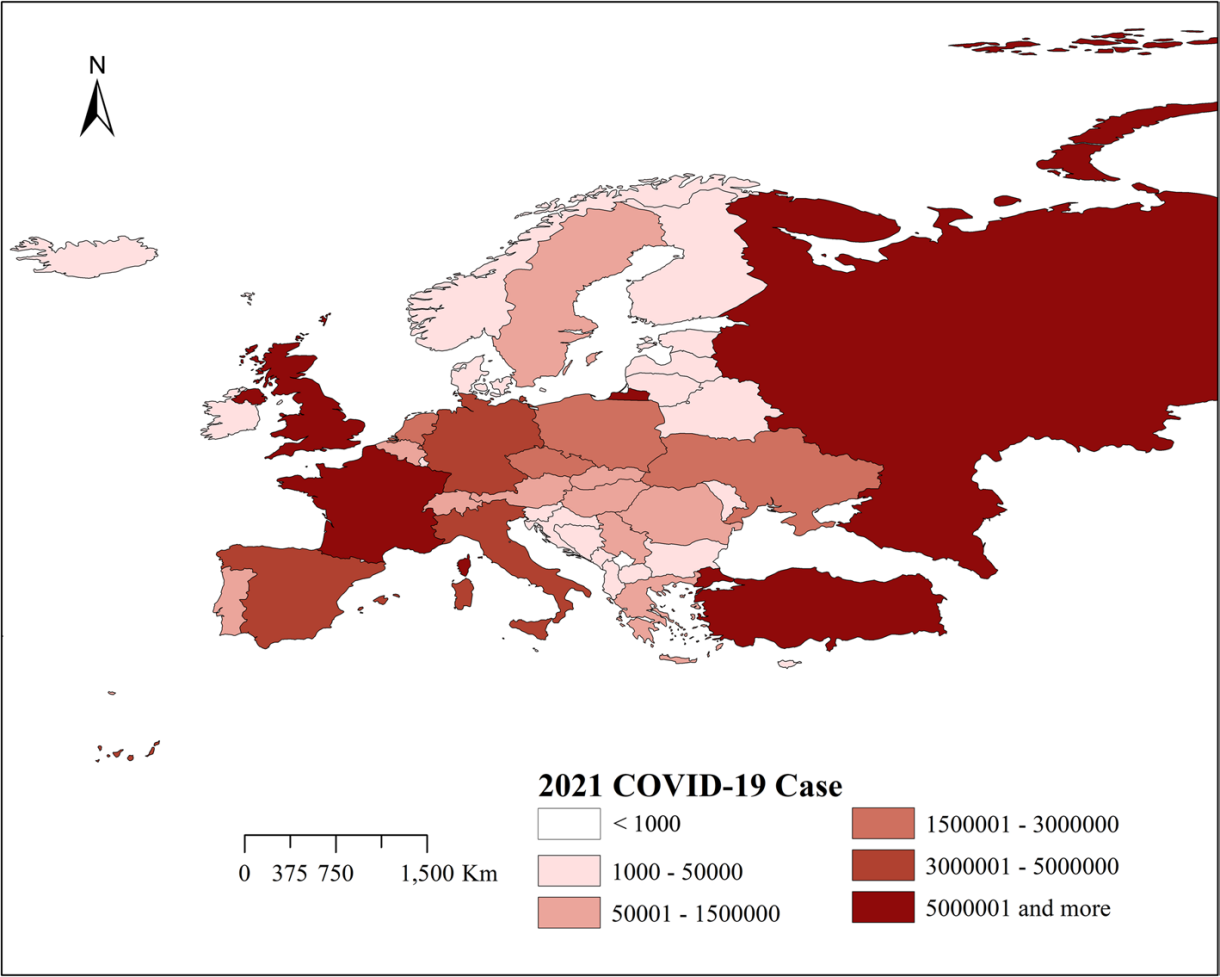
Spatial distribution map of COVID-19 cases in Europe between March 1st and October 2nd, 2021

Figure 3 shows the spatial distribution of the cumulative number of cases in 49 countries studied in Europe from March 1,2021 to October 2, 2021. The results show that among the 49 countries studied, the regions with the largest number of reported cases of COVID-19 are Western Europe, Central Europe, and Eastern Europe. The results in Fig. 1 show that among the 49 countries studied, the country with the largest cumulative number of reported cases is the United Kingdom (the darkest color), followed by Russia, Turkey, France, and Spain. The country (or region) with the lowest cumulative number of reported cases is the Faroe Islands.

### Space–time scan statistics

Table 3 shows the statistically significant space–time clusters of COVID-19 epidemics in European countries from March 1,2020 to October 2, 2020 and from March 1, 2021 to October 2, 2021.

**Table 3:** Space–time scan statistics

Cluster	Duration (days)	*P*	Observed	Expected	Observed / expected	RR	number of countries	countries RR > 1
2021(^*^Pro)								
1	Sep 2nd—Oct 2nd	0.001	4,635,765	730,668.15	3.09	3.65	1	1
2	Sep 25th—Oct 2nd	0.001	1,028,106	183,615.65	5.60	5.69	5	4
3	June 28th—Oct 2nd	0.001	3,162,801	1,871,254.78	1.69	1.74	1	1
4	Sep 3rd—Oct 2nd	0.001	284,648	73,807.01	3.86	3.87	3	3
5	Sep 2nd—Oct 2nd	0.001	263,274	83,029.50	3.17	3.18	1	1
6	June 30th—Oct 2nd	0.001	46,026	23,911.63	1.92	1.93	1	1
7	Sep 10th—Oct 2nd	0.001	3885	496.62	7.82	7.82	1	1
8	Sep 29th—Oct 2nd	0.001	65,527	50,178.13	1.31	1.31	7	3
9	July 15th – Oct 2nd	0.001	1196	763.97	1.57	1.57	1	1
2020 (^*^Pro)								
1	Sep 3rd—Oct 2nd	0.001	431,383	128,945.13	3.35	3.55	12	10
2	Aug 17th—Oct 2nd	0.001	448,381	172,882.69	2.59	2.74	3	3
3	Aug 6th—Oct 2nd	0.001	296,155	153,103.85	1.93	1.99	6	5
4	Sep 17th—Oct 2nd	0.001	130,271	46,023.60	2.83	2.88	5	3

Emerging space–time clusters of COVID-19 from March 1st-Octorber 2nd, 2020/2021 (*RR*   relative risk)

(^*^Pro prospective space–time scanning)

Table 4 shows the relative risk values of countries included in each COVID-19 space–time cluster from March 1, 2020 to October 2, 2020 and from March 1, 2021 to October 2, 2021.

**Table 4:** Location Relative Risk (RR = relative risk; LLR = log likelihood ratio; ID = country code)

Cluster	LLR	Radius(Km)	ID	Country name	RR	Observed	Expected
2021 Prospective Space–time Scanning							
Cluster 1	4,815,855.056913	0					
			47	Turkey	6.879505	4,635,765	730,668.15
Cluster 2	933,617.838604	697.63					
			30	Moldova	6.136766	56,083	9147.28
			48	Ukraine	6.92728	679,138	99,169.22
			38	Romania	1.633903	71,574	43,829.43
			7	Bulgaria	0.940797	14,830	15,762.95
			41	Serbia	13.195413	206,481	15,706.77
Cluster 3	385,591.008	0					
			49	United Kingdom	1.735884	3,162,801	1,871,254.78
Cluster 4	173,814.2676	403.91					
			46	Switzerland	3.877732	1294	333.71
			31	Monaco	3.867237	281,845	73,180.33
			40	San Marino	5.150877	1509	292.96
Cluster 5	123,893.247581	0					
			4	Belarus	3.182125	263,274	83,029.49
Cluster 6	8030.169858	0					
			9	Cyprus	1.925674	46,026	23,911.62
Cluster 7	4603.402691	0					
			2	Andorra	7.823446	3885	496.61
Cluster 8	2141.487015	508.78					
			3	Austria	0.775242	7824	10,091.87
			20	Hungary	0.563476	3487	6188.04
			42	Slovakia	0.167712	1858	11,076.52
			10	Czechia	0.242933	2946	12,124.61
			8	Croatia	1.347335	6199	4601.08
			43	Slovenia	1.82395	4334	2376.25
			6	Bosnia and Herzegovina	10.459311	38,879	3719.73
Cluster 9	104.032581	0					
			17	Gibraltar	1.565528	1196	763.96
2020 Prospective Space–time Scanning							
Cluster 1	227,282.141608	577.993412					
			26	Liechtenstein	0.719985	12	16.66
			46	Switzerland	1.5332	11,427	7458.48
			28	Luxembourg	1.21515	1918	1578.50
			43	Slovenia	3.600714	2932	814.59
			31	Monaco	2.609145	79	30.27
			40	San Marino	0.163412	17	104.02
			10	Czechia	4.798787	49,138	10,313.40
			8	Croatia	2.746837	6413	2336.43
			3	Austria	2.894494	18,576	6432.07
			5	Belgium	2.377368	42,424	17,925.92
			15	France	3.773486	291,498	80,415.52
			42	Slovakia	4.578696	6949	1519.19
Cluster 2	159,154.955163	502.317831					
			44	Spain	2.73704	447,119	172,346.96
			2	Andorra	2.376484	1061	446.50
			17	Gibraltar	2.25304	201	89.21
Cluster 3	54,294.662509	808.547268					
			48	Ukraine	2.436924	134,740	56,110.33
			30	Moldova	1.950873	28,250	14,517.43
			4	Belarus	0.50656	10,769	21,218.16
			38	Romania	2.153249	74,417	34,815.37
			27	Lithuania	1.974763	2647	1340.73
			36	Poland	1.812696	45,332	25,101.81
Cluster 4	51,955.958988	755.638764					
			22	Ireland	1.86288	5048	2710.94
			23	Isle of Man	0.039705	1	25.18
			49	United Kingdom	2.546001	85,950	34,085.78
			19	Guernsey	0.158199	3	18.96
			33	Netherlands	4.300221	39,269	9182.71

### The space–time cluster of COVID-19—March 1, 2020 to October 2, 2020

Cluster 1 is located in central and western Europe and contains 12 countries. A total of 431,383 cases have been observed. The cluster RR value is 3.55. Among them, the RR value of 10 countries is > 1, and the RR value of Czechia is the largest, which is 4.79. Cluster 2 is located in Western Europe and includes 3 countries including Spain. The cluster has observed 448,381 cases with an RR value of 2.74. The RR of the three risk countries are all > 1. Spain has the highest RR of 2.73. Cluster 3 includes 6 countries in Eastern Europe, with a cluster RR value of 1.99, of which 5 countries have RR > 1, and Ukraine has the highest RR value of 2.43. This cluster has a total of 296,155 observed cases. Cluster 4 is located in northwestern Europe. It has reported 130,271 cases in 5 countries. The cluster RR value is 2.88. Among them, 3 countries have RR > 1, and Netherlands has the highest RR of 4.30.

**Fig. 4:**
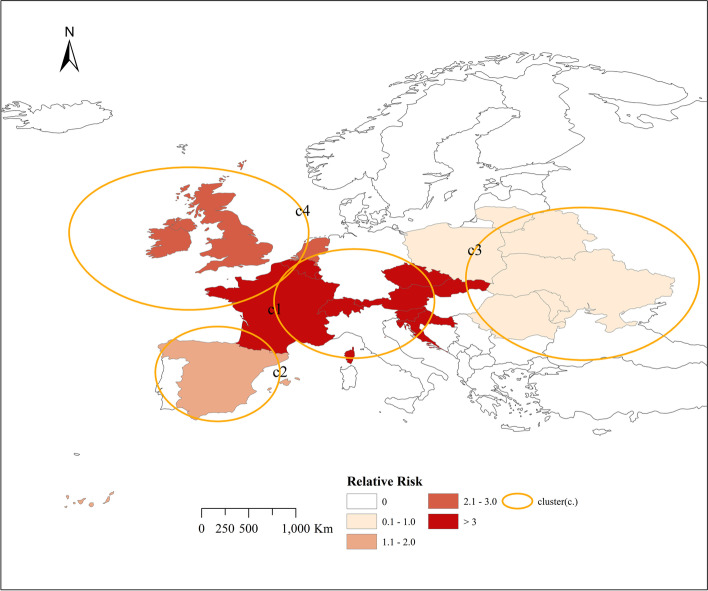
Spatial distribution of emerging space–time clusters of COVID-19 between March 1st and October 2nd, 2020

Figure 4 shows the location and spatial distribution of four statistically significant space–time clusters of the COVID-19 epidemic in Europe from March 1, 2020 to October 2, 2020, corresponding to the four clusters in the 2020 scan statistics in Table 3. Compared with Fig. 3, the results of the Poisson prospective space–time scan statistics are roughly the same as the actual results of COVID-19, which shows that the epidemiological statistical method is feasible. Therefore, we have made prospective results on the risk of the COVID-19 outbreak in Europe in 2021.

### Forecast analysis results from March 1st, 2020 to October 2nd, 2020

Table 5 shows the predicted values obtained by the statistical analysis of the prospective Poisson space–time scan from March 1st, 2020 to October 2nd, 2020, compared with the observed values in the same period in 2021, using the RMSE method.

**Table 5:** Root mean square error results analysis

observe cluster	observed value	predicted value	RMSE
cluster 1	128,946	128,945.13	1.6789
cluster 2	172,880	172,882.69	
cluster 3	153,105	153,103.85	
cluster 4	46,025	46,023.60	

(observed value: number of cases observed from March 1st, 2021 to October 2nd, 2021.)

### The space–time cluster of COVID-19—March 1, 2021 to October 2, 2021

Cluster 1 includes only one country, Turkey in Eastern Europe. At the time of this study, Turkey's RR is 3.65, with 4,635,765 observed cases. Cluster 2 contains some countries in Eastern Europe, and the cluster RR value is 5.69. There are 5 countries in total, of which 4 countries show RR > 1. They are Serbia (RR = 13.20), Ukraine (RR = 6.93), the country with the largest RR value., Moldova (RR = 6.14), Romania (RR = 1.63), 1,028,106 cases were observed. Cluster 3 only reported one country, the United Kingdom in northwestern Europe, with an RR of 1.74 and 3,162,801 observed cases. Cluster 4 is located in central Europe, with a cluster RR value of 3.87, including 3 risk countries which are San Marino (RR = 5.15), Switzerland (RR = 3.88), Monaco (RR = 3.87), with a total of 284,648 observed cases. Cluster 5 only reported one country, Belarus (RR = 3.18), located in Eastern Europe, with 263,274 observed cases. Cluster 6 also reported a country, Cyprus located in the northeast of the Mediterranean (Note: Although Cyprus belongs to Asia geographically, it is part of Europe historically, culturally, and politically, and is one of the countries in the European Economic Area), The cluster’s RR value is 1.93, and 46,026 cases were observed. Cluster 7 also contains only one country, Andorra, with an RR value of 7.82 and 3,885 observed cases. Cluster 8 is located in the central part of Europe and contains 7 countries. The cluster RR value is 1.31. Among them, 3 risk countries exhibit RR > 1, namely: Bosnia and Herzegovina (RR = 10.46), Slovenia (RR = 1.82), Croatia (RR = 1.35). Cluster 9 is located in a peninsula at the southern tip of western Europe. Only one area in Gibraltar is reported, with an RR value of 1.56, and 1,196 cases were observed.

**Fig. 5:**
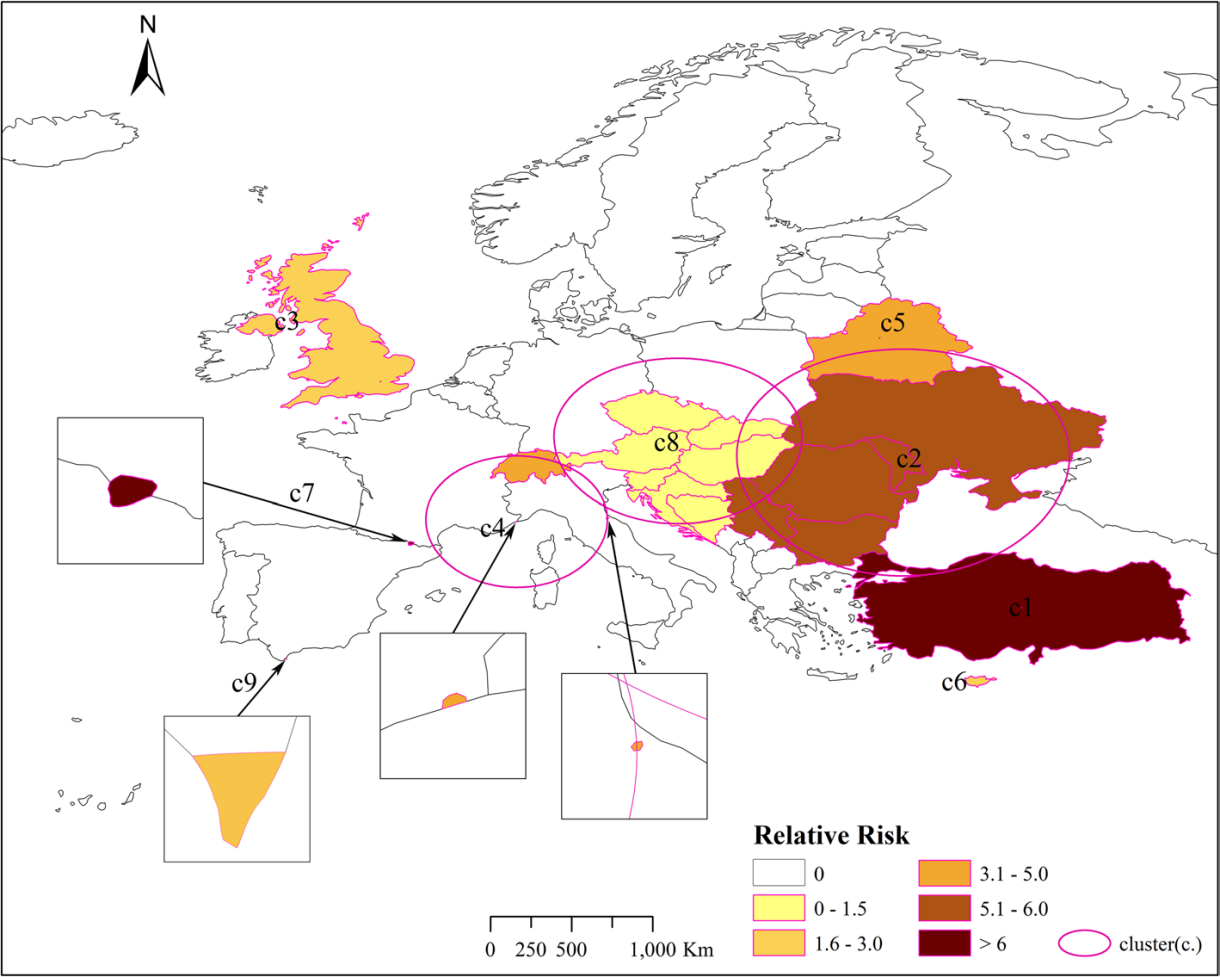
Spatial distribution of emerging space–time clusters of COVID-19 between March 1st and October 2nd, 2021

**Fig. 6:**
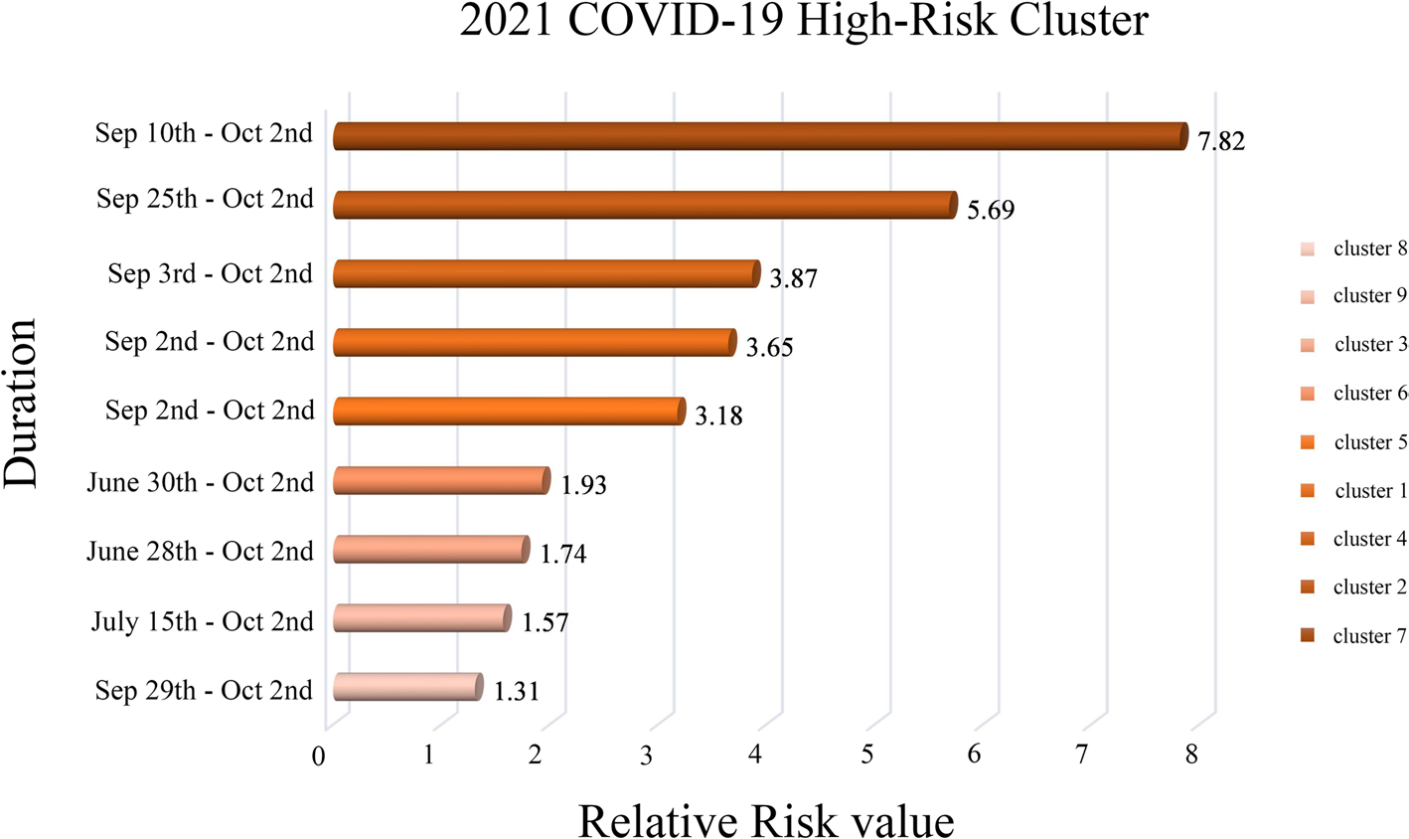
2021 high-risk clusters of COVID-19 in Europe

**Fig. 7:**
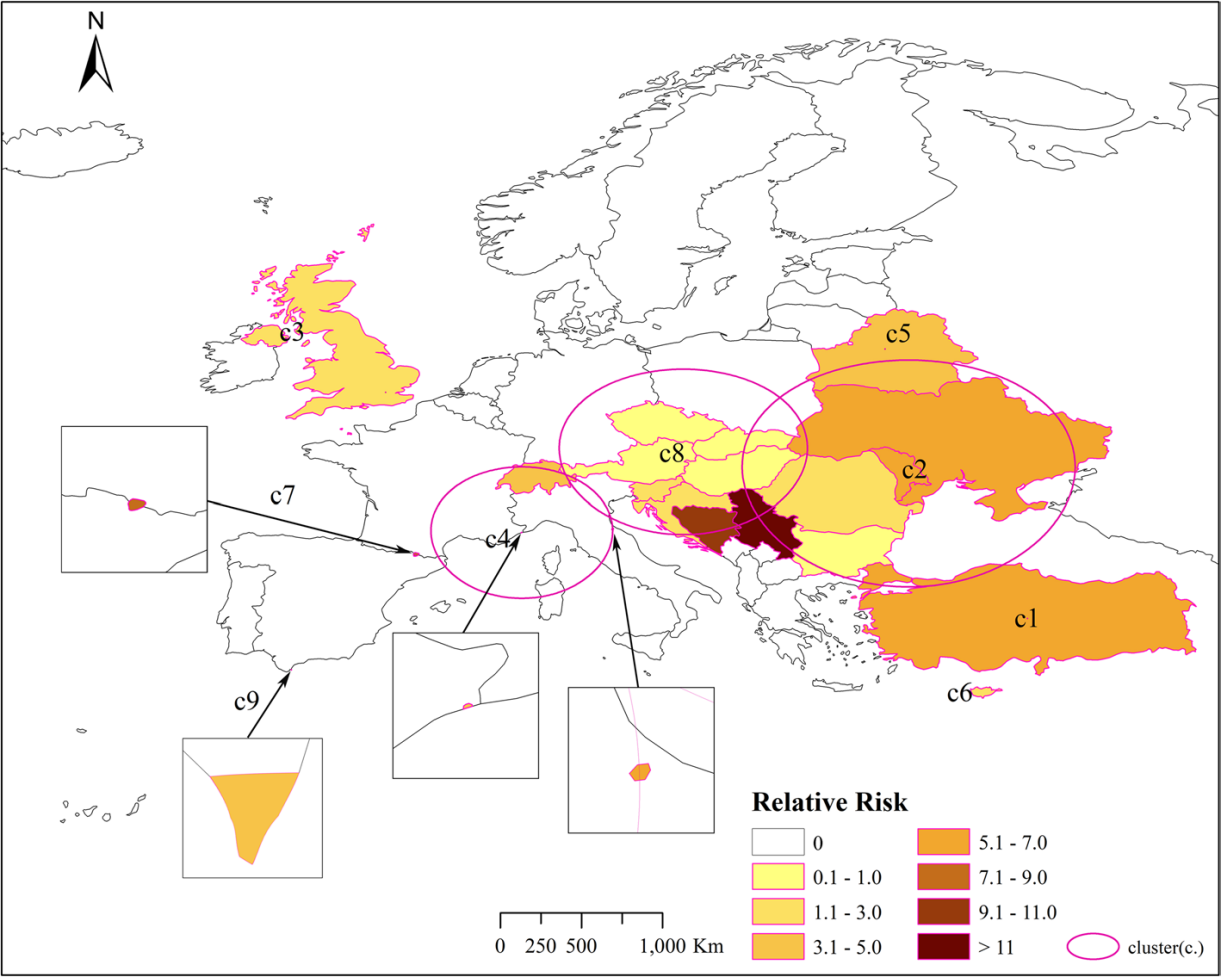
Spatial distribution of emerging space–time clusters of each country of COVID-19 between March 1st and October 2nd, 2021

**Fig. 8:**
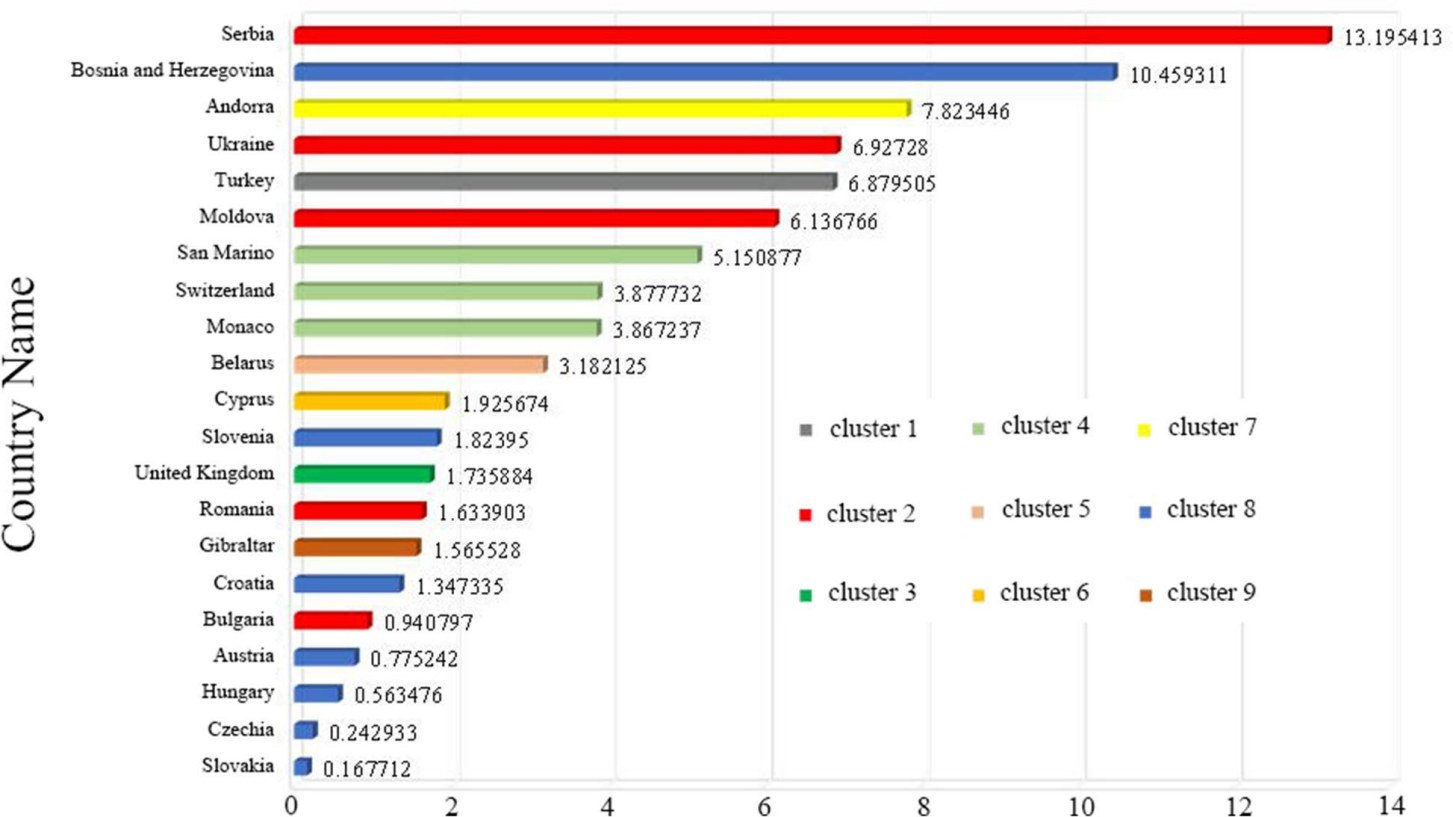
COVID-19 high-risk country description distribution in Europe in 2021

Figure 5 shows the location and spatial distribution of 9 statistically significant space–time clusters of the COVID-19 epidemic in Europe from March 1 to October 2, 2021, corresponding to the 9 clusters in the 2021 scan statistics in Table 3 (the specific numerical statistical results are shown in Fig. 6), including the 21 risk countries shown in Table 4 (the corresponding RR value statistical results of each country / region are shown in Figs. 7 and 8). The results show that the central and eastern regions of Europe are the center of COVID-19 in Europe. Countries with large populations are more likely to become high-risk areas, such as Turkey, the United Kingdom, and Ukraine. The results in Fig. 5 include some small-scale countries or regions, indicating that the definition of high-risk areas can be understood as areas with a high incidence of COVID-19, that is, more than 10% (20%) of the total population of the country or region. It is worth noting that Serbia is the country with the highest expected risk (Fig. 8). As shown in Figs. 3, 7 and 8, Moldova, Romania, Bulgaria, Switzerland, Andorra, and Belarus, which originally had a small cumulative number of cases, are all expected to be high-risk countries, indicating that the incidence rate of these countries is at a high level.

## Discussion

In this study, we used Poisson prospective space–time scan statistics to conduct space–time monitoring of COVID-19 in Europe. During the study period from March 1, 2020 to October 2, 2020 and from March 1, 2021 to October 2, 2021, we discovered emerging clusters of COVID-19 space–time clusters at the national level in the European region. In the prospective space–time scan statistics used in 2020, we set the maximum spatial scan area to 20% of the population at risk. This is because the scan results of other thresholds show that the number of countries included in some scan clusters is greater than the total number of countries studied 30% of the total number (> 14 countries), which leads to a significant reduction in the feasibility and effectiveness of disease surveillance. Secondly, the number of countries included in the scan results of other cutoffs exceeds 90% or more of the number of countries studied, and there are even repeated test results, indicating that the accuracy of clustering is too low and does not meet the actual situation of disease surveillance. This also shows that setting the maximum spatial scan area to 20% of the population at risk has the best fit.

When we analyzed the 2021 data set, we used the same method, but set the maximum spatial scan area to 10% of the population at risk. There are two main reasons: first, this is the setting after trying the previous method, and the result is similar to the previous analysis of the 2020 data set, that is, the number of countries included in some clusters exceeds 30% of the total number of countries studied or the results of repeated scanning appear, and the accuracy of the results is not high and does not conform to the actual situation. Second, we consider that the COVID-19 epidemic in 2020 is particularly serious, and more of it occurs in the form of outbreaks. The number of COVID-19 cases in various countries has suddenly increased, medical resources are scarce, there is no specific drug treatment, and human and material resources are insufficient. In response to the spread of COVID-19 at that time, and there was no vaccine developed at that time, so the 2020 data set analysis set 20% of the population at risk. On the contrary, China and the United States have developed the vaccine at the end of 2020. It is expected that in the past two years, people in most countries will be able to be vaccinated. Medical resources are sufficient, and human resources are increasing. Countries around the world have taken effective preventive measures, which effectively blocked the spread of COVID-19. Therefore, comprehensively weighing various situations, we finally set the maximum spatial scan area to 10% of the population at risk, and obtained the best fitting effect. The accuracy of cluster is relatively high, which is more in line with the actual situation of disease surveillance.

Combining the results of Figs. 3 and 4, the prospective space–time scan statistics from March 1, 2020 to October 2, 2020 reflect the actual incidence in 2021, in addition, the RMSE value shown in Table 5 in the results is 1.6789, which shows that the method we use is feasible. Through the setting of parameters, we have well predicted the actual situation of the COVID-19 epidemic in 2021, which also shows that our considerations are correct. It is precisely because of the better prediction effect in 2020 that we have made a prediction of the COVID-19 epidemic in 2021. Figure 5 shows that there will be the next wave of new COVID-19 epidemics in central and eastern Europe, of which country with the highest predicted relative risk is Serbia, followed by 21 countries including Bosnia and Herzegovina. This will provide public health decision-makers in relatively risky countries with information on the space–time development of disease outbreaks [18], prepare in advance for the prevention and control of the COVID-19 epidemic, strengthen restrictions on crowd movement, complete effective measures such as isolation and protection, and stop the re-eruption and spread of COVID-19.

Although our research has made some contributions, there are still some shortcomings and prospects. First of all, the prospective space–time scan statistics we use have certain limitations. It is a form in which the bottom scanning window is circular or elliptical. The scanning window is easily included in some surrounding areas that are not at risk. This makes the results have a certain error. In research areas with obvious spatial heterogeneity, a circle may be a bad choice [19]. This is very significant, because many of the clusters we have detected include some sea areas, which is obviously impractical. The solution to this problem is to change the circular or elliptical scanning window into an arbitrary shape. Flexibly shaped scan statistics [20] define the scanning window by connecting K-nearest neighbors to the focal area, which is especially suitable for detecting irregularly shaped clusters. Secondly, our data only includes the population, the number of confirmed cases, and the lack of subsequent potential infections. This result is largely the result of our testing work, and may not be a good representative of the real situation of the virus and the real space–time distribution. The only way to solve this phenomenon is to pass large-scale testing. Thirdly, when we apply the prospective space–time scan statistics method, the results of repeated scan statistics appear. This is the same as many statistics, and false positive results may eventually appear. But SaTScan™ provides a recurrence interval measurement, which quantifies the likelihood of accidentally observing clusters. We checked the recurrence intervals of our analysis and found that they were closely related to the p-values ​​we used to identify clusters. Prospective space–time scanning statistics have undeniable benefits for disease surveillance and are used by many public health agencies around the world [21]. Considering the recurrence interval of SaTScan™, it is recommended [22]. Fourth, we predict that the RR value of some clusters is not large, but there may be large risk differences within them, such as cluster 8 in 2021 in Table 3, where the relative risk span of 7 countries ranges from 0.1 to 10. This indicates that there are both high-risk countries and low-risk countries in some clusters. Local analysis of these countries or regions can provide a more accurate understanding of the counties or regions where the COVID-19 outbreak is at risk. Fifth, COVID-19 is more harmful to the elderly and people with pre-existing diseases. Our study did not use age and other related factors to correct. The results are not yet a good representative of the true situation of the overall population. Later studies can use the age-adjusted Bernoulli model to explain cases and deaths, while also adjusting other related factors. Sixth, our research is aimed at the national-level COVID-19 reports in the European region, and the accuracy needs to be improved. Furthermore, the number of countries we included in the study does not cover the entire European continent well, and there are certain errors in the results.

## Conclusion

We used open data from the European Centers for Disease Control and Prevention to detect emerging space–time clusters of COVID-19 in two different time periods in Europe. We suggest that emerging cluster countries with high RR and LLR values should pay attention to strengthen efforts in the implementation of national grass-roots monitoring and overseas imports, and take corresponding protective and quarantine measures in a timely manner to stop the spread and spread of COVID-19. Poisson prospective space–time scan statistical methods can effectively detect emerging clusters of COVID-19, and can monitor disease outbreaks when new data are available. In addition, we emphasize the importance of data sharing. During the COVID-19 pandemic, the availability of data sharing can monitor emerging and active clusters of cases with high accuracy, which is important for both regional decision makers and researchers. This can effectively use our epidemiological knowledge to effectively prevent and control the spread and spread of the COVID-19 epidemic, and provide sufficient space–time dynamic information and theoretical basis for public health decision-makers. Properly launch the implementation of epidemic prevention and control measures.

## Data Availability

The data is publicly available. The European Center for Disease Control and Prevention is an open access database. Researchers can access the relevant data set by logging in to https://www.ecdc.europa.eu/en/cases-2019-ncov-eueea. Data will be available on request by email to the corresponding author.

## References

[1] Dror AA, Eisenbach N, Taiber S (2020). Vaccine hesitancy: the next challenge in the fight against COVID-19. Eur J Epidemiol.

[2] Becker AD, Grantz KH, Hegde ST, Bérubé S, Cummings DAT, Wesolowski A (2021). Development and dissemination of infectious disease dynamic transmission models during the COVID-19 pandemic: what can we learn from other pathogens and how can we move forward?. The Lancet Digital Health.

[3] Ladoy A, Opota O, Carron PN (2021). Size and duration of COVID-19 clusters go along with a high SARS-CoV-2 viral load: A spatio-temporal investigation in Vaud state. Switzerland Sci Total Environ.

[4] Kulldorff M (1997). A spatial scan statistic. Communications In Statistics-Theory and Methods.

[5] Kulldorff M (2001). Prospective time periodic geographical disease surveillance using a scan statistic. J R Stat Soc Ser A.

[6] Jones RC, Liberatore M, Fernandez JR, Gerber SI (2006). Use of a prospective space- time scan statistic to prioritize shigellosis case investigations in an urban jurisdiction. Public Health Rep.

[7] Yin, F., Li, X., Ma, J., & Feng, Z. (2007). The early warning system based on the prospective space-time permutation statistic. Wei sheng yan jiu= Journal of hygiene research, 36(4), 455–458. 17953214

[8] Yih, W. K., Deshpande, S., Fuller, C., Heisey-Grove, D., Hsu, J., Kruskal, B. A., Kulldorff, M., Leach, M., Nordin, J., Patton-Levine, J., Puga, E., Sherwood, E., Shui, I., & Platt, R. (2010). Evaluating real-time syndromic surveillance signals from ambulatory care data in four states. Public health reports (Washington, D.C. : 1974), 125(1), 111–120. https://Doi.org/10.1177/003335491012500115. 10.1177/003335491012500115 PMC2789823 20402203

[9] Xu F, Beard K (2021). A comparison of prospective space-time scan statistics and spatiotemporal event sequence based clustering for COVID-19 surveillance. PLoS ONE.

[10] Rosillo N, Del-Aguila-Mejia J, Rojas-Benedicto A (2021). Real time surveillance of COVID-19 space and time clusters during the summer 2020 in Spain. BMC Public Health.

[11] Hohl A, Delmelle EM, Desjardins MR, Lan Y (2020). Daily surveillance of COVID-19 using the prospective space-time scan statistic in the United States. Spat Spatiotemporal Epidemiol.

[12] Tyrovolas S, Gine-Vazquez I, Fernandez D (2021). Estimating the COVID-19 spread through real-time population mobility patterns: surveillance in low- and middle-income Countries. J Med Internet Res.

[13] Desjardins MR, Hohl A, Delmelle EM (2020). Rapid surveillance of COVID-19 in the United States using a prospective space-time scan statistic: detecting and evaluating emerging clusters. Appl Geogr.

[14] Kulldorff M, Athas WF, Feurer EJ, Miller BA, Key CR (1998). Evaluating cluster alarms: a space-time scan statistic and brain cancer in Los Alamos, New Mexico. Am J Public Health.

[15] Kulldorff M (2007). A spatial scan statistic. Communications in Statistics - Theory and Methods.

[16] Ma Q, Gao J, Zhang W (2021). Spatio-temporal distribution characteristics of COVID-19 in China: a city-level modeling study. BMC Infect Dis.

[17] Xu M, Cao C, Zhang X (2021). Fine-scale space-time Cluster detection of COVID-19 in Mainland China using retrospective analysis. Int J Environ Res Public Health.

[18] Andrade LA, Gomes DS, Goes MAO (2020). Surveillance of the first cases of COVID-19 in Sergipe using a prospective spatiotemporal analysis: the spatial dispersion and its public health implications. Rev Soc Bras Med Trop.

[19] Takahashi K, Kulldorff M, Tango T, Yih K (2008). A flexibly shaped space-time scan statistic for disease outbreak detection and monitoring. Int J Health Geogr.

[20] Tango T, Takahashi K (2005). A flexibly shaped spatial scan statistic for detecting clusters. Int J Health Geogr.

[21] Greene SK, Peterson ER, Balan D (2021). Detecting COVID-19 Clusters at High Spatiotemporal Resolution, New York City, New York, USA, June-July 2020. Emerg Infect Dis.

[22] Kulldorff M, Kleinman K. Comments on 'a critical look at prospective surveillance using a scan statistic' by T. Correa, M. Costa, and R. Assuncao. Stat Med. 2015;34(7):1094–5. Doi:10.1002/sim.6430. 10.1002/sim.6430 PMC4357279 25754922

